# Community attitudes towards bears, bear bile use, and bear conservation in Luang Prabang, Lao PDR

**DOI:** 10.1186/s13002-019-0292-5

**Published:** 2019-02-26

**Authors:** Darunee Sukanan, Brandon P. Anthony

**Affiliations:** Central European University, Nádor u. 9, Budapest, 1051 Hungary

**Keywords:** Attitudes, Bear bile, Bear farming, *Helarctos malayanus*, Lao PDR, *Ursus thibetanus*

## Abstract

**Background:**

Bear bile is widely believed across much of Asia to have medicinal properties. As a result, great numbers of bears have been poached from the wild and numerous bear farms have been set up to drain the animals’ bile on a regular basis. Although most such farms are now illegal, they continue to exist in countries such as Lao PDR. A new bear sanctuary is under construction in Luang Prabang in the northern part of the country with the aim of providing shelter to bears rescued from these farms. Understanding the level and nature of local communities’ support for this sanctuary is vital for the long-term success of conservation efforts in the area, including outreach.

**Methods:**

This research, drawing from both ethnozoological and conservation frameworks, comprises a household survey (*n* = 263) administered in five villages surrounding the sanctuary and in-depth interviews conducted with key community leaders and institutional representatives. The questionnaire assessed local socio-economic status and attitudes towards bears, bear bile use, and bear conservation in general.

**Results:**

Respondents have generally positive attitudes towards bears and bear conservation. Age, gender, ethnicity, village, and household size have significant influence on attitudes towards bear bile use, which may also be determined by the expansion of sources for the supply of the traditional medicine market in neighboring China. However, many locals lack knowledge about the current status of wild and captive bears. This may be due to inadequate outreach involving community incentives for positively influencing attitudes. We argue that local communities will need to be integrated into conservation efforts while enhancing knowledge of conservation issues through improved outreach and communication.

**Conclusion:**

Positive attitudes towards bears appear prevalent in the communities surrounding the new sanctuary. Villagers are familiar with laws regarding wildlife conservation but lack a deeper understanding of the status and plight of wild bears in the country, particularly how bear farming is a threat to the species. Conservation efforts must entail culturally relevant co-educational initiatives to garner further support from local communities.

## Introduction

The relationships between fauna and human physical and cultural evolution likely predates historical evidence and has been manifested in a wide variety of ways, including the utilitarian role of both wild and domesticated animals as food, labor, pets, ornaments, and medicine [1]. The study of these relationships, ethnozoology, has gained prominence in recent decades as researchers and practitioners alike have sought meaning to the use of animals by human societies, particularly its contribution towards declining wildlife populations [1, 2]. More specifically, the use of animals in traditional medicine systems is also gaining increased attention [3, 4], particularly in China where more than 1500 animal species having traditional medicinal use have been recorded [5]. What is less well known is how medicinal use is transmitted across countries and cultures, a sub-theme of ethnozoology which seeks to understand the complexity and implications of human-animal interactions [2].

The Malayan sun bear (*Helarctos malayanus*, Raffles 1821) and Asiatic black bear (*Ursus thibetanus,* G. [Baron] Cuvier 1823), also popularly known as the “moon bear”, are found across much of South and Southeast Asia, including Lao PDR. Both species are listed as “vulnerable” by IUCN because their overall populations have declined by 30% [6] and 30–49% [7], respectively, over the past 30 years and continue to decrease. The dramatic declines in both species have been caused by a reduction of suitable habitats, coupled with widespread exploitation for body parts for traditional medicine, including paws and bile [8].

Bear bile has been used since at least the Tang Dynasty in traditional Chinese medicine and has proven medicinal properties [9, 10]. However, alternatives to bear bile, including synthetic bear bile and others originating from non-threatened animals and plants, have been demonstrated and introduced [10, 11]. Yet the consumption of, and international trade in, bear bile has proven resistant to such efforts [12], with bears being poached regularly across their range. Addressing this conservation challenge has been multifaceted and calls for understanding across a range of disciplines including conservation biology and ethnozoology [1].

### Bear farms

In response to increasing threats, bear farms have been established to extract bear bile non-lethally in the hope that farmed animals can serve as more sustainable sources of bile for medicinal use and reduce demand for poaching of wild bears [12, 13]. However, the growth of bear farms in China, South Korea, Vietnam, Myanmar, and Lao PDR [7, 14] has seen a concomitant increase in the number of bears being illegally sourced from the wild or internationally imported [15, 16]. Factors influencing this illegal market include the fact that bears caught from the wild are often cheaper and easier to obtain than breeding bears at farms where poor conditions are commonplace [17], and many consumers consider products obtained from wild animals as more potent than from farmed animals [18, 19].

Bear farms operated mostly by Vietnamese nationals were established in Lao PDR after bile extraction at farms in Viet Nam was banned in 2006 [16, 20]. The number of captive bears in Lao PDR increased from 40 in 2008 to 122 in 2012, some of which were being housed at illegally operating breeding facilities [15, 18]. A high mortality rate was observed at some of these farms, suggesting that captive bears were likely brought from the wild as replacements for those that perished in captivity [16, 21].

### Bear sanctuaries

The hunting of moon and sun bears as well as the possession of their parts is prohibited in Lao PDR by the Wildlife and Aquatic Law (2007), although Article 40 of the same legislation allows endangered species, including bears born in captivity as second-generation animals, to be traded with permission. At the 17th meeting of the Conference of the Parties to CITES (CoP17) in 2016, the government of Lao PDR committed to shutting down tiger and bear bile farms in the country in part due to increasing criticism of widespread illegal farm practices [22]. Free the Bears Fund Inc. (FTB), an international conservationist group, seeks to help eradicate all bear bile farming in Lao PDR by 2020 and has stepped up efforts to establish sanctuaries to house bears rescued from farms [23].

The Luang Prabang Wildlife Rescue Center (LPWRC) is a new bear sanctuary under construction in Luang Prabang, Lao PDR, in cooperation between FTB and the Luang Prabang Provincial Agriculture and Forestry Office. The sanctuary, expected to house up to 150 bears, is located in a rural and non-touristic area approximately 8.5 km away from an existing bear sanctuary (Tat Kuang Si Bear Rescue Center, hereafter TKSBRC) [24].

The total project site is 24.8 ha. The area will be divided into 6 separate bear enclosures with varying sizes, of which the total area is 6.6 ha, including a 0.88 ha quarantine area and five separate enclosures ranging from 0.85 ha to 1.5 ha.

As Asiatic black bears are omnivores, efforts are made to ensure a varied diet that resembles their natural diet. The food provided to the bears primarily consists of fruits, nuts, tubers, and vegetables, in addition to treats such as dollops of honey. The food is usually delivered in ways, such as hidden inside enrichment tools, that force the bears to forage for them within their enclosures.

FTB plans to increase locals’ appreciation of the animals through building awareness of the bears’ nature and habits. One such effort entails allowing local people to visit the facility so that they can learn the individual stories and personalities of captive bears housed at the sanctuary, as well as at another similar site nearby.

### Cooperation with local communities

Many local communities in developing countries, including Lao PDR, depend on and utilize natural resources which in turn demand more nuanced conservation strategies to prevent overexploitation. Enlisting the support of local people and integrating their participation to conserve wildlife are considered crucial to sustainable and effective conservation management and efforts [25–28].

The attitudes of local people towards wildlife and its conservation can be strongly impacted by demographic and socio-cultural factors including interrelationships with target species [2] and the management and governance of conservation efforts [29], as well as perceived socio-economic improvements provided by a protected area [30–32]. Conversely, negative attitudes can be triggered when local people consider the services and benefits provided by conservation efforts to be too costly, including when experiencing damage by protected wildlife [4, 33], when resource access is restricted [29], or when local culture is ignored [34]. Thus, understanding these multidimensional factors influencing people’s attitudes is critical to achieving sustainability and efficiency in wildlife conservation.

A recent study exploring the potential disparities between public perceptions by local Lao people and Chinese visitors pertaining to the use of bear parts showed that foreign Chinese respondents tended to have higher awareness of cruelty inflicted on captive bears, while local Lao respondents tended to lack sufficient knowledge about the actual situation of bears in Lao PDR, including their declining numbers in the wild [12]. The study also suggested that conservation campaigns in Lao PDR should be aimed at improving the knowledge of locals about the plight of bears in the country.

As there is currently no research on the performance of bear conservation efforts in the Luang Prabang area, and as LPWRC is still under construction, we aimed to contribute to the expanding ethnozoological field by investigating local knowledge and attitudes on bears and their conservation, bear bile use, and bear sanctuaries, which can be used as a benchmark for future monitoring and evaluation at LPWRC, as well as other sanctuaries in relevant contexts in Lao PDR or elsewhere. Moreover, we seek to deepen understanding of the dynamic relationship between our target population and its evolving relationships with the perceived health benefits from threatened bear species [35].

## Materials and methods

### Study area

**Fig. 1:**
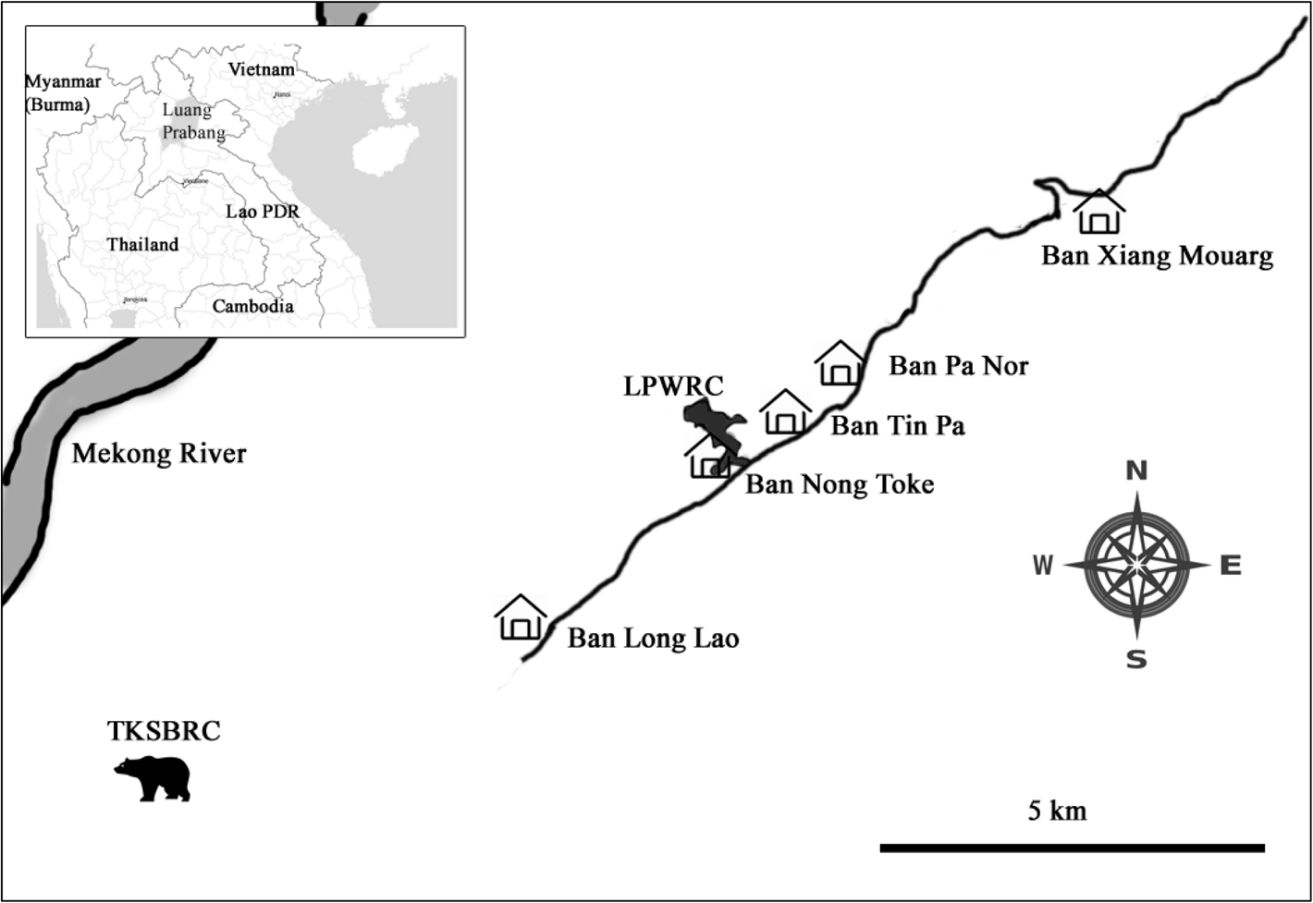
Location of the old sanctuary, TKSBRC, the new sanctuary, LPWRC, and the five sampled villages

Field research was carried out in May 2017 in Luang Prabang Province, located in the mountainous north of Lao PDR. Five rural villages (Xiang Mouarg, Pa Nor, Tin Pan, Nong Toke, Long Lao) are proximally located to the sanctuary along a single unpaved road that extends from Luang Prabang and terminates at Long Lao (Fig. 1). Nong Toke and Tin Pa share boundaries with LPWRC, and land that has been utilized to construct the new bear sanctuary was bought from these two communities. Livelihoods in the area are primarily agriculturally based, with both crop cultivation and livestock husbandry practiced.

People who live in the area comprise three ethnicities: Lao, Khmu, and Hmong. The Lao and Khmu are predominantly Buddhists, whereas many Hmong are animists. The Khmu and Hmong have their own distinct languages, however Lao is the country’s official language that most people from every ethnic group can speak to varying degrees and use routinely to communicate with members of other ethnic groups.

Residents in Nong Toke and Tin Pa are cognizant of the LPWRC project as there have been village consultation meetings, while those from other nearby villages learned of the ongoing project via awareness signs erected on the main road.

### Field methods and data collection

A mixed-methods approach was used for collecting both quantitative and qualitative data.

#### Household questionnaire

In seeking to gauge the attitudes of local people towards wildlife conservation, attitudinal surveys can produce important data that assist conservationists in understanding local attitudes, perceptions, and value orientations that have a bearing on their behaviors [25, 36] . Although attitudes do not always accurately predict behavior [37], they can be confidently used to predict certain behaviors that can impact local conservation efforts [38, 39]. A face-to-face questionnaire was administered to individuals ≥ 18 years old from 263 randomly selected households (60.2% of all households; CI = 3.82, CL = 95%) in all five villages. The Lao Women’s Union of Luang Prabang, which had previously worked with villagers in these communities, assisted in obtaining the required prior permission from each village head. During the process, information about the researcher, purpose, and content of the survey were relayed to each village head, who were then requested for permission for the survey to be conducted before the surveyors could proceed. The questionnaire consisted of closed-ended questions exploring the following: (1) demographic characteristics, (2) socio-economic conditions, and (3) attitudes and beliefs towards bears, bear bile use, and wildlife sanctuaries. The questionnaire was originally written in English before being translated into Lao by a professional translator; subsequently, the text was translated back into English by another professional translator to check if any inconsistencies in translation had occurred inadvertently. It was pre-tested on 10 local Lao people to ensure that the questions were understood as intended. As educational levels among Lao villagers vary, ranging from functional illiteracy to university degrees, some questions were subsequently modified to ensure that respondents with different levels of education understood them consistently.

Throughout the entire research process, Central European University’s (CEU) Ethical Research Guidelines and Ethical Research Policy were adhered to. Five assistants from the Lao Women’s Union of Luang Prabang were recruited for collecting data as they had experience in conducting similar research surveys with FTB. Assistants were trained and each was asked to sign a declaration form that they would not engage in any form of coercion with participants and would adhere to our ethics protocol. Before each interview, assistants verbally informed respondents concerning the research aims, purpose of the survey, and asked for verbal consent.

#### Representative interviews

Semi-structured interviews were carried out with the heads of the five villages who serve as official community representatives, in order to elicit data that might be overlooked or could not be acquired from questionnaires alone [40]. These interviews consisted of questioning along four lines: (1) bears and bears in the wild, (2) bear bile use, (3) the existing TKSBRC, and (4) the new bear sanctuary (LPWRC). Our ethical research protocol was adhered to including verbal consent to participate, anonymity, and confidentiality. The interview was conducted in Thai and interviewees responded in Lao, a cognate language. In addition, semi-structured interviews in English were conducted with an FTB representative concerning the nature of the organization’s public relations outreach projects and bear management policies.

### Data analysis

All quantitative data acquired from the household survey were analyzed using SPSS v22.0 [41]. Measures of central tendency and dispersion were calculated based on the nature of the variable and normality of the data. Significant correlations and differences across participant responses were sought according to six independent variables (age, gender, village, number of assets, ethnicity, persons/household). Tests for correlation (Pearson’s *r*, Spearman’s rho) were based on the nature of the tested variables. For categorical variables, chi-square (*χ*^2^) was accompanied with the adjusted standardized residual (asr) of the item, indicating the direction and standard deviation from the mean [42]. Our null hypotheses are that there are no significant differences across our independent variables with respect to bear conservation knowledge, bear bile use, efficiency of various medical traditions, and attitudes towards bears and bear sanctuaries. Alpha was set at 0.05.

Inductive analysis was used to analyze qualitative data acquired from our semi-structured interviews [40]. Results of interviews with village heads were categorized into the four themes explored, while data provided by FTB representatives were categorized under the rubric of public relations outreach projects and bear management policies.

## Results

### Socio-demographic features of respondents

**Fig. 2:**
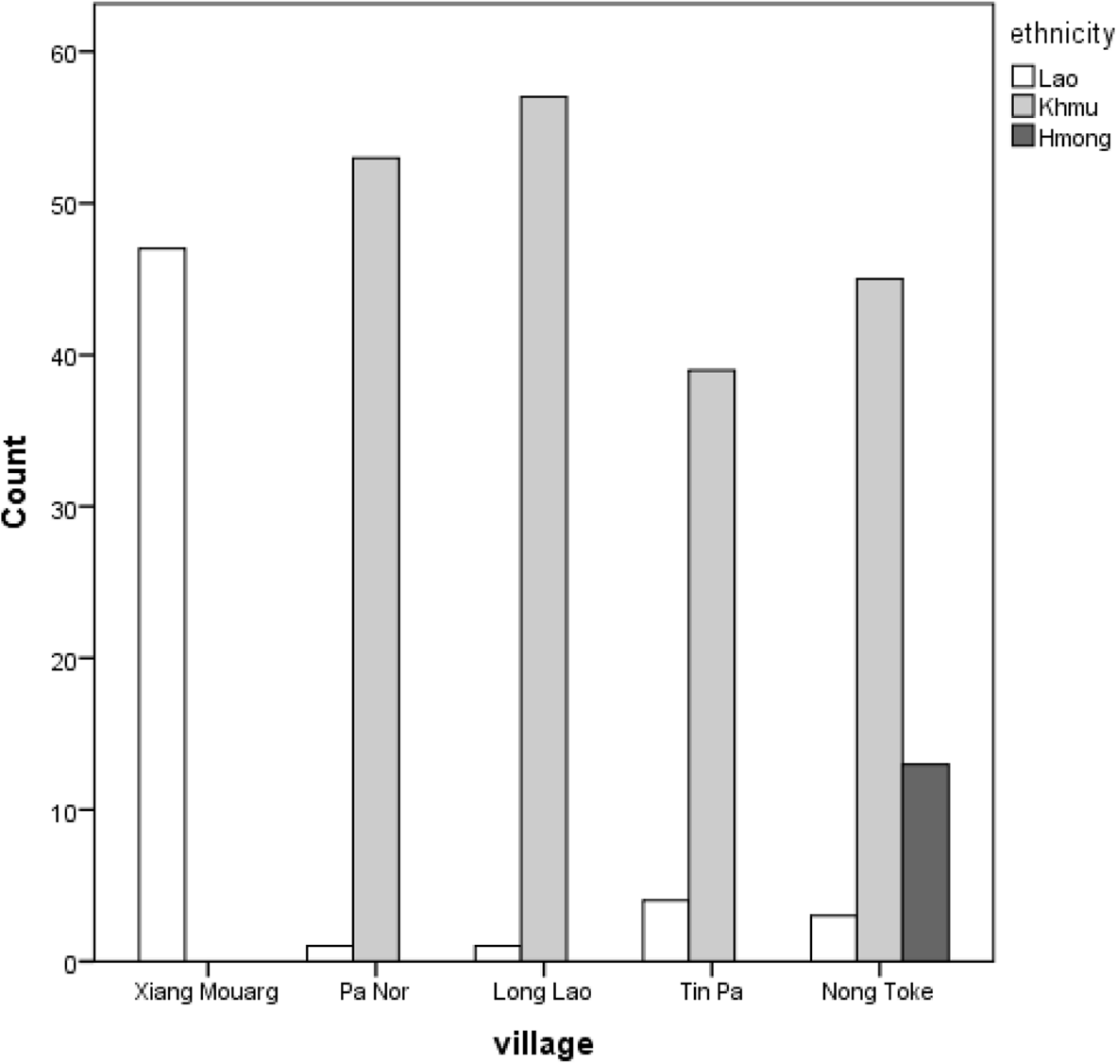
Distribution of survey respondents across village and ethnicity (*N* = 263)

A total of 263 respondents were surveyed from five villages, of whom, 114 were male (43.3%) and 149 female (56.7%). Respondent mean age was 45.7 years (sd = 13.82), and ranged from 18 to 82 years. Most survey respondents were Khmu (73.8%), while Lao and Hmong people accounted for 21.3% and 4.9%, respectively (Fig. 2). Most survey participants were either household heads (46.8%) or spouses of household heads (46.8%). The number of people per household ranged from 1 to 14 (median = 5, skewness = 0.756).

#### Socio-economic status of households

Households had a mean of 2.58 rooms (sd = 0.750) and ranged from 1 to 6. Although most households (87.2%) have access to electricity, the main source of energy for cooking (96.2%) is wood due to the relative high cost of electrical power. Sources of household water were primarily natural water bodies (89.0%), followed by piped water (6.5%) and boreholes or wells (4.5%). Education levels among our respondents varied, with 8.1% of households having all members functionally illiterate; 5.0% claiming that at least one family member had attained literacy certificates and partial completion of primary school; 80.3% stating that at least one family member had attained high school education, while 6.6% had a family member with at least a bachelor’s degree.

To assess household asset ownership, respondents were asked if they possessed the following 13 assets: tractor, car or van, motorbike, bicycle, boat, radio, television, landline telephone, mobile phone, computer, air conditioner, fan, and fridge or freezer. The number of these assets per household ranged from 0 to 9 ($\overline{x}$ = 4.6, sd = 1.81) and was positively correlated with number of persons per household (*r* = 16, *p* < .01, *n* = 261). Moreover, ANOVA and Scheffe post-hoc tests revealed that household asset number significantly varied according to village (*F* = 9.956, df = 4, *p* < .001), with Xiang Mouarg having significantly higher assets ($\overline{x}$ = 5.77, sd = 1.108) than Tin Pa (mean diff. = 2.022, p < .001), Long Lao (mean diff. = 1.611, *p* < .001), and Nong Toke (mean diff. = 1.356, *p* < .01), and Pa Nor having significantly higher assets ($\overline{x}$ = 4.91, sd = 1.804) than Tin Pa (mean diff. = 1.163, *p* < .05).

There are various elements that contribute to variation in relative affluence among the villages. According to interviews with village heads, the main factors include the following:

1. Occupation: Although most villagers are farmers, in Ban Xiang Mouarg, most families were relatively more affluent because many residents there operate as middlemen who buy produce from farmers in other villages and sell it at a higher cost in the city.
2. Land size: Each village has its own territory, which has varying sizes. As a result, in some villages, many residents hold larger parcels of land available for growing and harvesting crops than others.
3. Village chiefs’ policies: The relative affluence of residents in each village depends in part on their chief because of the often top-down nature of decision-making. If a village chief has policies in place such that every household should possess its own land for agriculture and keeping livestock, villagers in that community tend to be more affluent, and vice versa. For example, some villages sold pieces of their land to outside buyers, which contributed to impoverishment in those villages.

### Attitudes and beliefs towards bears, bear bile use and wildlife sanctuaries

Most respondents (82.5%) said that they had seen bears and many had seen them only at the TKSBRC. Based on a 5-point Likert scale, 70.7% of respondents stated that they like wild bears, however 7.6% indicated they did not, while 21.7% neither liked or disliked them or were unsure. Liking bears was positively correlated with household size (rho = 0.177, *p* < .01, *n* = 261), age (rho = 0.117, *p* < .05, *n* = 254), and number of assets (rho = 0.137, p < .05, n = 261) and was significantly influenced by village (${X}_8^2$ = 22.062, *p* < .01), in which Tin Pa had significantly lower number of respondents who indicated they “liked” bears (asr = − 3.1).

Approximately 74% of respondents expressed that they liked that wild bears existed in Lao PDR, while 6.1% disagreed with this statement. Similarly, this factor was positively correlated with age (rho = 0.174, *p* < .01, *n* = 254) and household size (rho = 0.163, *p* < .01, *n* = 261). Further, this factor was significantly influenced by village (${X}_8^2$ = 21.719, p < .01), whereby Pa Nor respondents were more likely to dislike bears in the wild than expected (asr = 3.0), while, conversely, respondents from Long Lao were more likely to like bears in the wild than expected (asr = 2.4).

Even though most participants (55.3%) indicated they did not know whether there were more or fewer bears than in the past in Lao PDR, 30.9% believed that the bear population was increasing, while 13.7% believed it was decreasing (Table 1). There was also a high percentage of respondents (84.7%) who knew that hunting bears in Lao PDR was illegal, and 8.4% who believed this activity was legal, with the remaining 6.9% unsure. Although many respondents (46.2%) did not believe that bear bile could be extracted without killing bears, 29.4% recognized that this method was possible. A large number of respondents (71.8%) believed that most farm bears were born in captivity, whereas only 4.2% did not believe so. Most participants (63.4%) believed that consuming bear bile in Lao PDR was illegal, whereas 9.5% responded that it was legal.

**Table 1:** Respondent beliefs (as %) concerning bear conservation and legislation, and valued opinions concerning bear bile use (*n* = 262; where applicable, correct responses are highlighted in bold)

Statement	True	False	Do not know
Bear conservation and legislation			
The number of bears in Laos is increasing	30.9	**13.7**	55.3
It is possible to extract bile from a bear without killing the animal	**29.4**	46.2	24.4
Most bears in farms were born in captivity	71.8	**4.2**	24.0
Hunting bears in Laos is legal	8.4	**84.7**	6.9
Consuming bear products in Laos is legal	9.5	**63.4**	27.1
Valued opinions concerning bear bile use			
Most people whose opinion I value have used bear bile for medicine and other purposes in the past	42.7	15.3	42.0
Most people whose opinion I value will continue using bear bile in the future	34.0	8.8	57.3
Most people believe you should use bear bile	32.4	17.6	50.0

We tested for significant differences among correct responses to these knowledge statements across our six independent variables and found a number of notable cases (Table 2). Gender, ethnicity, village and household size were each found to significantly contribute to correct responses.

**Table 2:** Tests for significant correlations between belief statements and independent variables

Statement	Age	Gender	Ethnicity	Village	hh size	Assets
Bear conservation and legislation^a^						
The number of bears in Laos is increasing (false)	ns	Cramer’s *V* = .214** (male)	Cramer’s *V* = .378*** (Hmong)	ns	*F* = 3.086* (greater)	ns
It is possible to extract bile from a bear without killing the animal (true)	ns	ns	ns	ns	ns	ns
Most bears in farms were born in captivity (false)	ns	ns	ns	ns	*F* = 14.411*** (lower)	ns
Hunting bears in Laos is legal (false)	ns	ns	ns	Cramer’s *V* = .173* (Long Lao)	*F* = 9.010*** (greater)	ns
Consuming bear products in Laos is legal (false)	ns	ns	Cramer’s *V* = .291*** (Khmu)	Cramer’s *V* = .228** (Long Lao)	ns	ns
Valued opinions concerning bear bile use^b^						
Most people whose opinion I value have used bear bile for medicine and other purposes in the past	ns	Cramer’s *V* = .197** (male)	Cramer’s *V* = .208*** (Hmong)	ns	ns	ns
Most people whose opinion I value will continue using bear bile in the future	ns	ns	Cramer’s *V* = .179* (Lao)	Cramer’s *V* = .175* (Xiang Mouarg)	ns	ns
Most people believe you should use bear bile	ns	ns	Cramer’s *V* = .192** (Lao)	Cramer’s *V* = .248*** (Xiang Mouarg)	ns	ns

^a^significant differences shown for orientation towards correct responses [indicated in square brackets following statement], compared with other groups

^b^significant differences shown for orientation towards “true” responses, compared with other groups

*ns* not significant, *p < .05, **p < .01, ***p < .001

Almost half the respondents (42.7%) valued people who had used bear bile for medicinal and other purposes in the past and would use it in the future (34.0%) (Table 1). Almost twice as many respondents indicated that most people believe one should use bear bile than those who did not think so, although 50% of our sample were unsure. Again, we tested for significant differences towards personal orientation with these statements across our independent variables (Table 2) and found that gender, ethnicity, and village were all contributing factors.

**Fig. 3:**
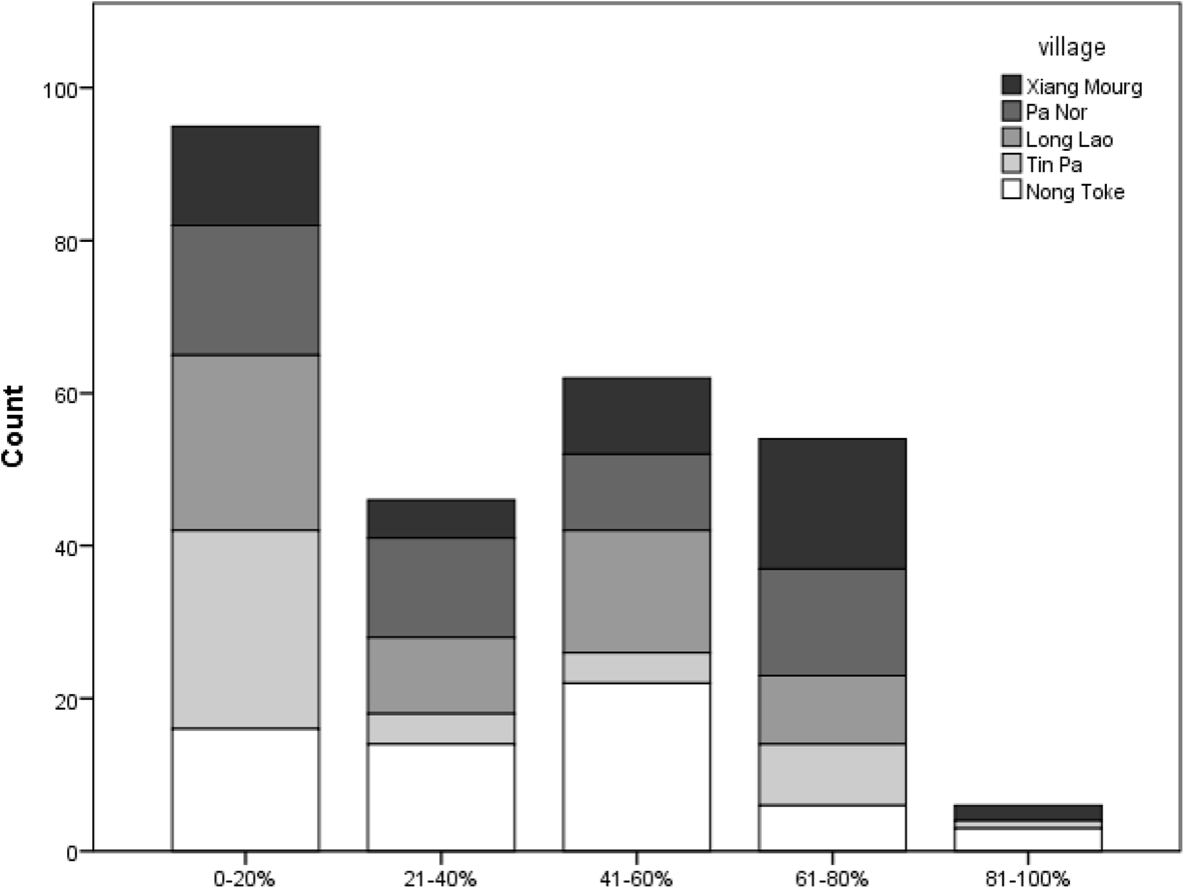
Estimation of percentage of friends and family members who use bear bile/bear bile products, according to village (*N* = 263)

Moreover, a significant number of respondents thought that many of their closest relatives and friends might use bear bile and bear bile products: close to one quarter thought that 41–60% of their friends and relatives might use bear bile, while one in five thought that most (61–80%) of their friends and relatives might do so (Fig. 3). There were significant positive correlations between increased proportion of friends and relatives believed to be using bile with age (rho = .166, *p* < .01, *n* = 254) and household assets (rho = .142, *p* < .05, *n* = 263). Moreover, village was a significant factor (${X}_{16}^2$ = 40.058, *p* < .01) with respondents from Tin Pa having higher than expected counts in the 0–20% range than other groups (asr = 3.6) and Xiang Mouarg with higher than expected counts (asr = 2.9) in the 61–80% category.

**Fig. 4:**
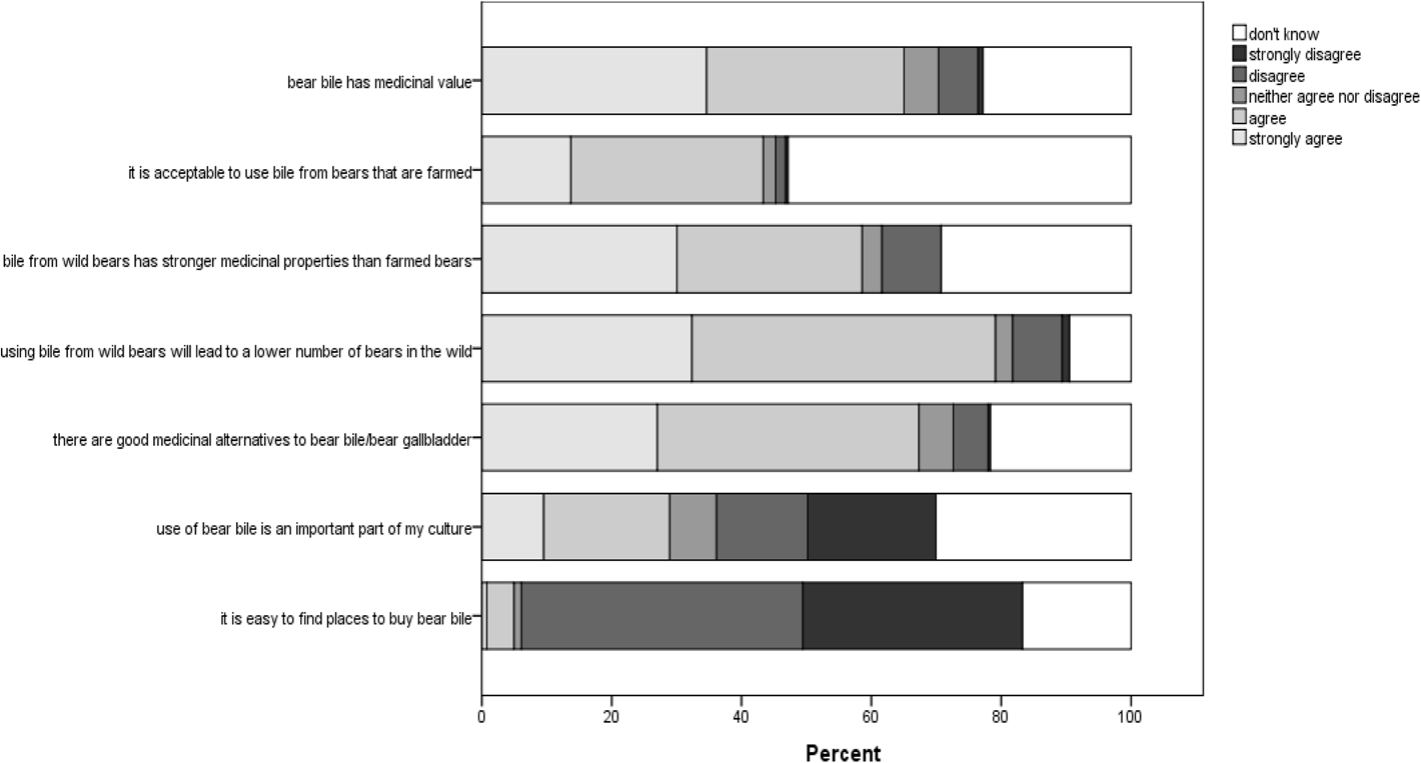
Distribution of opinions concerning bear bile use (*N* = 263)

Most participants (65.0%) believed that bear bile has medicinal value (Fig. 4), with 28.9% agreeing that it was an important aspect of their culture. Even though a high percentage (79.1%) of respondents was aware that consuming bear bile would lower the number of bears in the wild, many (43.7%) expressed the view that consuming bear bile from animals kept at farms was acceptable. Moreover, close to 60% of respondents believed that bear bile obtained from wild bears had higher medicinal value than bile obtained from farmed bears, and 67.3% thought that there were good alternative medicines to bear bile and gall bladder. Finally, most respondents (77.1%) said they believed that bear bile was not readily available. Responses to these Likert-type scales were recoded to collapse all responses to either “agree” or “disagree”, excluding others. These were then tested for independence across the six independent variables (Table 3). All independent variables had significant effects on level of agreement with at least one statement, with age, village, and household size, having more widespread influence across all statements.

**Table 3:** Tests for significant correlations between agreeing with statements and independent variables

Statement^a^	Age	Gender	Ethnicity	Village	hh size	Assets
Bear bile has medicinal value	ns	Phi = .163* (female)	ns	ns	*t* = 2.086* df = 185	ns
It is acceptable to use bile from bears that are farmed	*t* = 2.198* df = 113	ns	ns	ns	ns	ns
Bile from wild bears has stronger medicinal properties than farmed bears	ns	ns	Cramer’s *V* = .193* Khmu (asr = −2.68)	Cramer’s *V* = .300** Pa Nor (asr = − 3.8)	*t* = 2.567** df = 175	ns
Using bile from wild bears will lead to a lower number of bears in the wild	*t* = 1.836* df = 222	ns	ns	Cramer’s *V* = .229* Tin Pa (asr = −3.3)	*t* = 2.610** df = 228	ns
There are good medicinal alternatives to bear bile/bear gallbladder	ns	ns	ns	ns	ns	t = 1.917* df = 190
Use of bear bile is an important part of my culture	*t* = 2.332* df = 159	ns	ns	ns	ns	ns
It is easy to find places to buy bear bile	ns	ns	ns	Cramer’s *V* = .266** Nong Toke (asr = 3.3)	ns	ns

^a^significant differences shown for orientation towards “agree” responses, compared with other groups

*ns* not significant, *p < .05, **p < .01

A strong majority of respondents indicated they “highly valued” environmental conservation, including that carried out by the Lao Forestry Service (92.8%), conservation workers (92.8%), and the TKSBRC (88.6%).

#### Local views on the efficiency of western and traditional medicine

Respondents were asked to indicate to what extent they valued authorities on medicine. A vast majority (91.3%) stated that they “moderately” to “highly” value western medical experts. This was positively correlated with household size (rho = .270, *p* < .001, *n* = 261) and number of assets (rho = .107, *p* < .05, *n* = 263) and significantly influenced by village (${X}_{12}^2$ = 39.213, *p* < .001). In this case, Long Lao were more likely to “moderately” to “highly” value western medical experts than expected (asr = 2.7), while Tin Pa respondents were less likely to do so (asr = − 5.5).

Similarly, a large majority of respondents (86.3%) indicated that they “moderately” to “highly” value religious leaders, which was positively correlated with age (rho = .133, *p* < .05, *n* = 254). Moreover, it was strongly affected by ethnicity (${X}_6^2$ = 116.783, *p* < .001), whereby Khmu respondents were more likely to “moderately” to “highly” value religious leaders than expected (asr = 4.3), while Hmong were less likely to (asr = − 9.3). Similar to value ascribed to western medical experts, valuing religious leaders was also significantly influenced by village (${X}_{12}^2$ = 44.123, *p* < .001). In this case, Long Lao were more likely to “moderately” to “highly” value religious leaders than expected (asr = 3.4) while Tin Pa and Nong Toke respondents were less likely to do so (asr = − 2.0 and − 3.3, respectively).

Lastly, most respondents (71.5%) stated that they “moderately” to “highly” value traditional healers, a trait which was significantly held by the Hmong (${X}_6^2$ = 17.525, *p* < .01).

More than half of our respondents (56.3%) thought that a combination of western and traditional medicines was the most effective treatment for ailments. This information corresponds to the forms of medicine that respondents had used to treat ailments from which they had suffered in the previous 12 months: 81.4% of all respondents had used western medicine and 45.1% had used traditional herbal curatives to treat their ailments, with a mere 0.8% using synthetic bile and 0.8% farmed bile (*n* = 262).

### Insights from community representatives

In order to gain insights that could not be acquired from the survey, village heads from the five villages were interviewed during hour-long face-to-face meetings.

#### Attitudes towards bears and bears in the wild

When asked how they felt about wild bears and efforts to house rescued bears nearby at a newly constructed sanctuary, some village heads contended that bears were dangerous and thus had to be kept safely away from people:

Bears eat people, so the sanctuary [LPWRC] should have all safety systems in place to protect people.

I have never seen wild bears here, only at Tat Kuang Si [existing bear sanctuary]. I am afraid of bears. They attack people. I have heard it said that before, maybe a hundred years ago, there were many bears and many attacks on people. However, for the past 40-50 years, I have never heard about a case that a bear attacked people.

However, all interviewees also held positive attitudes towards wild bears, acknowledging the important role they play in ecosystems and concern over their decreasing numbers.

Bears are good for the environment. They are important part of it. In the past, there was a lot of wildlife, but now there are very few bears.

One bear was found near here last year. I was delighted that there were still some wild animals [like that] in the forest.

I like bears because it is hard to see them. Only if you go to see bears at Tat Kuang Si or the zoo in Vientiane [the capital of Lao PDR].

#### Attitudes towards bear bile use

When representatives were asked their opinion regarding the medicinal properties of bear bile, responses ranged widely and can be categorized into four intensities.

1. Unsure

I have never seen any villagers using bear bile. I heard somebody say it had medicinal potential but I do not know how good it is and I do not know whether it has real medicinal value or not.

2. No medicinal value

Personally, I do not think that bear bile is good for curing diseases. You have to go and see a doctor to check for diseases.

3. Some medicinal value

Bears are valuable animals. They have [parts that are] potent medicines.

Bear bile is good for medicine but I do not know whether it is better than western medicine because I have never used it to cure diseases. However, one of my friends brought it to me to give it a try and I felt good; I felt that my body was lighter.

4. High medicinal value

People who have more money want to get bear bile. It is the best medicine among all others, including western medicine. People who do not have money will have to choose normal medicine. Some diseases can be cured only by bear bile.

Pure bear bile or bear bile that is not mixed with anything has more medicinal value than western cures. It also depends on what types of diseases people have. Bear bile can be more effective in curing thalassemia [a blood disorder] than western medicine.

One respondent also commented that his local village does not use bile due to high cost, difficult to access, and doubt over authenticity.

Bear bile is not good for us because, firstly, it is very expensive and, secondly, it is very hard to find and, thirdly, we do not know whether what they are selling is real bear bile. We do not buy bear bile. It is better to go and see a doctor.

The representatives were subsequently asked their knowledge of, and experience with, bear farms. Some were unaware of their existence; however, others stated they knew about bear farms but had personally never visited any. Those who had knowledge concerning bear farms were then asked for their opinions on these farms. Representatives were divided in this regard, some supporting the idea of bear farms with others disapproving of their usefulness.

#### Attitudes towards tat Kuang Si bear rescue center (TKSBRC)

Village heads were invited to offer their opinion of the TKSBRC. All five showed positive attitudes towards the sanctuary, citing the various forms of income mobilization it has generated including tourism, employment, local development, and sale of local produce for bear food. In addition, some also supported the existing bear sanctuary for non-economic reasons, i.e., its objective and efforts to conserve bears.

I agree with [people at] Tat Kuang Si because they take care of bears rescued from traffickers … Sometimes wild animals such as bears and tigers are seized from smugglers at the boundary between Laos and Vietnam.

#### Attitudes towards LPWRC

In order to investigate representatives’ attitudes towards LPWRC, they were first asked whether they thought the new bear sanctuary would bring benefits to people in the villages. Responses revealed three primary reasons for supporting the LPWRC. First, they expect to receive economic benefits from increased domestic and foreign tourists, resulting in sustaining local livelihoods and improved infrastructure.

Many tourists will come to visit the sanctuary. Money from tourists can be used for developing local villages.

Benefits that the locals might get are that they can get some incomes from selling vegetables for bear food. Tourists also will come here and buy some produce from the locals.

I am happy that the government decided to build the new bear sanctuary here. People will have more jobs and they can improve their financial situation. People here are poor. We want to help them get out of poverty.

The roads will be improved. The locals can sell their produce or they can build accommodations for rent. I am glad and hope everything will improve.

A second reason that representatives offered was that the sanctuary would help preserve the animals, even if locals might not personally benefit from the bears.

Even though the locals cannot get benefits or get bear bile from the bear sanctuary, it is still a good effort because it will help conserve bears.

It is good if there are conservation efforts to save wildlife and bears. Firstly, there are no [wild] bears in Luang Prabang. Secondly, children can go to see bears. They have never seen wildlife. They have only heard of them.

If there are organizations who conserve wild animals that have almost gone extinct, I agree with them. Nowadays, there are not even deer or wild pigs in the forest here.

A final reason that representatives gave for endorsing the new sanctuary was that locals would be able to source bear bile from there in future, indicating that at least some village heads misunderstood the actual purpose of the sanctuary.

The advantages the villagers may get from the bear sanctuary are firstly that they can ask to buy bear bile from the sanctuary.

I’m happy that in the future this can be a sanctuary that will produce many bears and lots of bile. The government may have a policy to distribute bear bile to cure diseases. It might happen in future.

Representatives were subsequently asked what negative impacts the new bear sanctuary might generate for locals and which should be considered in the planning phase. Some raised concerns that because bears are regarded as dangerous animals, breakages out of the facility by bears, or children sneaking into the facility, may result in bear attacks on people. A second concern related to the risk of zoonoses that could potentially be spread by bears brought from other areas. Further, one interviewee expressed concerns that unpleasant odors might emanate from the bear enclosures. Finally, a lack of communication and interaction between LPWRC and nearby communities was identified as a disturbing factor by some village heads, which was felt to potentially affect the acceptance of the facility by villagers.

It is good to have conservationists working here but I would like them to come and inform the locals in detail about the animals they are going to conserve; how they keep the animals and what advantages or disadvantages there will be for villagers. For example, if bear conservationists inform me about these details, I as a village chief can inform my people about the advantages and disadvantages of the new sanctuary. Otherwise, we should warn our kids not to go near the place.

#### FTB public relations outreach projects and bear management policies

When asked whether FTB had policies that would help ensure that LPWRC enjoyed increased cooperation from local people to help conserve bears, the group’s representative stated that FTB would offer free guided tours in LPWRC for local people "… to build awareness and connection between people and the animals", to "show them the bears, tell them their stories, tell them about their personalities and how smart they are" in order to raise awareness of conservation issues pertaining to bears and foster increased appreciation for the animals. He also emphasized the prevailing differences in commonly held views between people in the West and Asia concerning wildlife and its conservation and that FTB plans that any outreach policies should be culturally relevant to local villagers.

You have to recognize the culture differences between the west and Asia. There is a very utilitarian mindset here [whereby locals] treat wildlife as sources of food or medicine, whereas people in western nations [have generally] moved further away from living in close proximity to wildlife and have as a result become more affectionate towards wildlife. [People in the West] see something on TV [about wild animals], they read something in books. They see wildlife in a controlled way. They do not see any direct negative impact on their way of life. But here we know bears go into farms so they can create human-bear conflicts. We would like to see a growth in affection towards bears but you have to be aware that is not necessarily the ultimate goal. It’s enough if local people just understand that these bears are predators in the wild where they have lived for many generations and that if people continue to hunt them they won’t be around much longer. So that may be a more effective way of talking to people and encouraging them to help conserve bears.

The FTB representative also stated that he did not expect any future conflicts because LPWRC had plans to address community concerns, including (1) erecting reliable electric fencing, (2) establishing secure quarantine areas for new bears to address disease risk, and (3) creating meaningful employment for locals and engaging in other income-generation projects such as purchasing produce (bananas, cucumbers, other vegetables) from locals for bear food. Upon reflection, however, he added that the existing TKSBRC had become a source of conflict with local people over complaints that water from the center drained into and contaminated local water sources. In response, channels were installed to collect water that drained from the sanctuary and the waste water was treated before being released into natural water resources. He indicated similar troughs would be built in LPWRC to prevent water from the new sanctuary polluting local sources.

## Discussion

### Local attitudes towards bears and environmental conservation

In the surveyed communities, although villagers recognized bears as potentially dangerous predators [43–45], most liked bears and their national presence, particularly those who were older and from larger, more affluent households. This positive orientation may be a result of the scarcity of any direct negative interaction with wild bears as most had been limited to viewing them in the controlled setting of TKSBRC [46] or because affluent households were better situated to economically benefit from the presence of the sanctuary. Moreover, positive attitudes towards environmental conservation in general was also manifest, indicating that villagers had experienced minimal negative impacts from conservation efforts and understood the need for such efforts to continue. Building on such positive attitudes towards bears and environmental conservation is of vital importance, which would be best served with educational, community outreach and awareness-raising initiatives in order to boost local people’s appreciation of endangered wild animals and foster better understanding of the purpose and practice of conservation [47, 48]. Such initiatives could leverage the positive role that older and more affluent people in villages hold, particularly in offering free guided tours at the new sanctuary and educational workshops pertaining to bears and conservation as per the plan suggested by FTB.

### Public perceptions regarding bears and bile farming

We demonstrate that knowledge concerning bear hunting and bile consumption laws is widely evident. Less well-known aspects of bear conservation, however, are a concern, including the decreasing population trends of wild bears in Lao PDR. This awareness is shaped by socio-demographic factors, which should be considered in awareness raising initiatives. Our results confirm findings by Davis et al. [12] that increasing knowledge among locals of the declining number of bears in the wild in Lao PDR should be a priority for any conservation campaign.

The nature of bear farming practices was inadequately understood by members of the communities. Very few respondents, including some village heads, believed that it was possible to obtain bear bile without killing bears, indicating unfamiliarity with the actual practice of bile farming. This may be due to the infancy of bear farming practices in Lao PDR, which only gained momentum after 2006 [15, 49]. In contrast to recent studies that show that many bears raised at farms had been illegally taken from the wild or had been imported from outside the country [15, 17], our study indicates a widespread belief that most bears kept at farms were born in captivity. Therefore, it would seem essential that villagers receive accurate, up-to-date information about the dramatically declining number of bears in the wild as well as bear farming practices [10], in addition to how such practices not only harm the wellbeing of captive animals but also pose a tangible threat to extant wild bear populations in Lao PDR.

### Views on bear bile use

There was widespread consensus in our study that reinforced commonly held views of the benefits of using (especially wild) bear bile [18, 19], but again, the perceived medicinal value was determined by inter- and intra-village differences, indicating that relationships between bear bile and these communities are not ubiquitous and may be open to change. This perceived value of bear bile may be decreasing for a number of reasons.

First, less than half of our respondents stated that significant people they know had ever used bear bile, and any use would likely decrease in the future. Second, only a minimal proportion of our respondents believed the use of bear bile was an important part of their culture, suggesting its use may be driven by other factors and that any cultural norms pertaining to the importance of bear bile use may be on the decline, which can be expected as the knowledge system of any culture is not static but is a dynamic process of assimilating “outside” knowledge and synthesizing and hybridizing existing knowledge [50, 51]. This finding corroborates those of Davis et al. (2016) [12], suggesting that unlike in China where bear bile use has long been seen as an integral part of traditional medicine [10], the increase in bear bile production in Southeast Asia has been caused by sudden high foreign demand rather than by local culture, especially in countries bordering China, including Lao PDR and Myanmar [18]. Third, as most respondents believe good medicinal alternatives to bear bile and bear gall bladder exist, and very few reported using either synthetic or farmed bear bile, we believe that most people in these communities do not place an inordinately high value on the alleged medicinal properties of bear bile. If alternative medicinal treatments become more available and affordable in the area, including more remote villages where experienced doctors are scarce [52], beliefs in the efficacy of bear bile may de-intensify further, which will aid conservation efforts.

Despite its recognized properties, it is difficult to find places where locals can buy bear bile. One reason for this could be that locals are generally familiar with the wildlife laws in Lao PDR that forbid possessing, hunting, and capturing wild bears. Related to its scarcity, bear bile is also viewed as an expensive traditional medicine that only more affluent families can afford.

Our results extend the previous exploration of community attitudes towards bear bile use by investigating the effects of a number of demographic and socio-economic factors. We show that demographic and socio-cultural factors significantly influence attitudes towards bear bile use. Older respondents were more likely to recognize that bile sourced from wild bears are decreasing their population, accept the use of bile from farmed bears, and agree that the use of bear bile was an important part of their culture. This finding is similar to that in neighboring Viet Nam which showed that older people tend to be bear bile consumers as a result of more traditional ways of thinking and increased reliance on traditional medicine [53]. Compared to men, women respondents were more likely to agree that bear bile had medicinal value, which appears counter-intuitive as men were more likely to use bile than women in Quyen Thi’s [53] study. However, their findings varied among location, which involved only urban respondents, while ours was restricted to rural villages, and belief in the medicinal value of bile is not necessarily correlative with use. Tailored interventions that leverage pro-conservation attributes within these local communities should be sought; for example, working with local leaders who share pro-conservation views and behaviors [54] and the exploration of suitable substitutes for bear bile as a medicine [9–11].

Finally, as expected, households with a greater number of assets were more likely to agree that good alternatives to bear bile exist, presumably as such households would have greater economic flexibility in obtaining these products. However, as our study is novel with respect to these factors, delineating these differences in a follow-up study would be useful.

### Attitudes towards traditional and western medicine

Bear bile has been consumed widely in Southeast Asia, and elsewhere, as an ingredient in traditional medicine to treat a variety of ailments and diseases because beliefs in the potency of animal parts continue to persist and people continue to ascribe healing properties to certain body parts, including bear bile [10]. Although western medicine is increasingly prized by locals, reliance on traditional medicine persists, particularly in rural areas within Lao PDR where western medicinal practices have not yet fully penetrated [55, 56]. This coincides with our study, although reported bile use was negligible. As it is widely known that the consumption of bear products is illegal in Lao PDR, we recognize that our results may have underestimated the true proportion of bile use by our respondents, as fear of retribution and social desirability bias in such contexts can be high [57]. This is also supported by the relatively higher proportions of highly valued friends/family members respondents claimed had used, will use, or claim one should use bear bile. The uneven adoption of bear bile as traditional medicine in our studied communities is noteworthy from an ethnozoological perspective as, presumably, these communities have not had a long tradition with bear bile, only gaining increased exposure to it as foreign demand from China triggered a growth in bear farms in the country. Our study sheds light on how human cultures are dynamic and their relationship with wild animal species may be driven by socio-economic factors, even those from abroad. This is an important finding which needs to be investigated further as this cultural fluidity may be evident in other contexts where sourcing declining wildlife populations from new areas may also have reciprocal effects by creating (new) local demand for such wildlife, particularly as traditional medicine. This is disconcerting, as such demand may exacerbate unsustainable exploitation of threatened species and contribute to their decline in novel ways. From an ethnozoological perspective, it would be prudent for researchers to monitor this dynamic relationship whereby human-animal interactions, sensu consumptive uses, expand and contract in response to changing socio-economic and cultural factors [58].

### Link between perceived incentives provided by LPWRC and attitudes

Our study shows that local communities entertained more positive attitudes towards bear conservation because they expected socio-economic benefits from the newly built sanctuary. This is based not only on the observed tangible benefits accruing to villages located next to the existing TKSBRC, but also to the proposed measures by LPWRC. These findings align with other studies showing that financial incentives provided by protected areas to nearby communities can lead to positive attitudes [24]. The communities surrounding LPWRC are considered “rural” [59] and most residents are farmers. According to village heads, villagers expect LPWRC to provide them with new and improved sources of income, including gainful employment, tourism revenues, and the sale of produce as bear food. In addition, they also expressed hope that the new sanctuary would lead to better infrastructure in the area. Yet, despite a number of village meetings and road signage, inadequate communication and interaction between LPWRC and nearby communities was observed by some village heads, which may adversely impact attitudes towards LPWRC. For instance, some villagers expressed hope, despite the stated policies of LPWRC, that they might now be able to obtain a steady supply of bear bile from the new sanctuary. They also expressed concern that animals kept at LPWRC could lead to human-bear conflicts and bring new diseases to the area.

However, FTB has wisely anticipated such misunderstandings. During the period of our study, plans were underway to build quarantines for keeping bears brought from other areas, the construction of bear-proof electric fencing, and wastewater treatment channels, as well as anticipating income generating initiatives for local villagers. Such community expectations of meaningful involvement and socio-economic improvement are critically important and need to be heeded because failing to do so may jeopardize the local acceptance and legitimacy of the sanctuary because "… lack of interaction, poor communication, [and] unfulfilled promises in terms of financial compensation … can create confusion and mistrust with respect to the purposes of a protected area and its alleged commitment to improve relationships with its neighbours" [4].

## Conclusion

Our study contributes to the growing ethnozoological literature and is the first to investigate the role of demographic and socio-economic factors in explaining knowledge and attitudes towards bears and their conservation, bear bile use, and bear sanctuaries in Lao PDR. The plight of sun and moon bears, in many respects, depends on their importance to those sectors of society which value their use in traditional medicine [3]. We demonstrate that beliefs, attitudes, and practices towards bears and bear bile use are not universal and vary according to demographic and socio-economic factors. As a result, bear conservation efforts must entail culturally-relevant co-educational initiatives to garner further support from local communities.

Further incentives provided by LWPRC, especially those that enhance livelihoods, are likely to generate more favorable attitudes towards conservation efforts. Because of their relatively isolated location in an underdeveloped rural area, the sanctuary’s neighboring communities expect to benefit significantly from the creation of new jobs, diversified income revenues from tourists and tourism-related activities, sale of local produce, and development of new infrastructure. Such improvements will, however, necessitate not only a firm sustained commitment to meeting these obligations, but also enhanced outreach and closer collaboration between the sanctuary and neighboring communities. In parallel, continuing the examination of the role of bear bile as traditional medicine in the area is critical, and how this use is linked with changing social-ecological systems.

Our findings have direct utility for FTB and conservation interventions in/around the newly constructed LPWRC bear sanctuary. At the same time, however, they elucidate the ethnozoological complexities that conservation in such contexts can present. With such interventions, community expectations become manifest, which such endeavors must plan for, and appreciate, in order to succeed. In our case, the fate of two threatened bear species stands in the balance.

## Abbreviations

FTB: Free the Bears Fund Inc.
LPWRC: Luang Prabang Wildlife Rescue Center
TKSBRC: Tat Kuang Si Bear Rescue Center

## References

[1] Alves RRN (2012). Relationships between fauna and people and the role of ethnozoology in animal conservation. Ethnobiol Conserv.

[2] Alves RRN, Souto WMS, Albuquerque UP, Alves RRN (2018). Ethnozoology: conceptual and historical aspects. Ethnozoology: animals in our lives.

[3] Alves RR, Rosa IL. Why study the use of animal products in traditional medicines? J Ethnobiol Ethnomed. 2005;1(5). 10.1186/1746-4269-1-5. 10.1186/1746-4269-1-5 PMC1277085 16270931

[4] Anthony BP (2007). The dual nature of parks: attitudes of neighbouring communities towards Kruger National Park, South Africa. Environ Conserv.

[5] Yinfeng G, Xueying Z, Yan C, Di W, Sung W. Sustainability of wildlife use in traditional Chinese medicine, conserving China’s biodiversity: reports of the Biodiversity Working Group (BWG). China Council for International Cooperation on environment and Development. 1997:190–220.

[6] Fredriksson G, Steinmetz R, Wong S, Garshelis DL, (IUCN SSC Bear Specialist Group) (2008). Helarctos malayanus. The IUCN Red List of Threatened Species 2008.

[7] Garshelis D, Steinmetz R (2016). Ursus thibetanus. The IUCN Red List of Threatened Species 2016.

[8] Mills J, Servheen C (1994). The Asian trade in bears and bear parts: impacts and conservation recommendations. Bears Biol Manage.

[9] Wang DQ-H, Carey MC (2014). Therapeutic uses of animal biles in traditional Chinese medicine: an ethnopharmacological, biophysical chemical and medicinal review. World J Gastroenterol.

[10] Feng Y, Siu K, Wang N, Ng K, Tsao S, Nagamatsu T (2009). Bear bile: dilemma of traditional medicinal use and animal protection. J Ethnobiol Ethnomed.

[11] Li S, Tan HY, Wang N, Ming H, Li L, Cheung F, et al. Substitutes for bear bile for the treatment of liver diseases: research progress and future perspective. Evid Based Complement Alternat Med. 2016:4305074. 10.1155/2016/4305074. 10.1155/2016/4305074 PMC4819118 27087822

[12] Davis EO, O’Connor D, Crudge B, Carignan A, Glickman JA, Browne-Nunez C (2016). Understanding public perceptions and motivations around bear part use: a study in northern Laos of attitudes of Chinese tourists and Lao PDR nationals. Biol Conserv.

[13] Damania R, Bulte EH (2007). The economics of wildlife farming and endangered species conservation. Ecol Econ.

[14] Education for Nature-Vietnam (ENV) (2010). An analysis of attitudes and bear bile use in Vietnam.

[15] Actman A. Inside the disturbing world of bear bile farming. Natl Geogr. 2016. Available at: https://news.nationalgeographic.com/2016/05/160505-asiatic-bear-bile-trade-laos/. Accessed 5 May 2016.

[16] Foley KE, Stengel CJ, Shepherd CR (2011). Pills, powders, vials and flakes: the bear bile trade in Asia.

[17] Hance J. Is the end of ‘house of horror’ bear bile factories in sight? The Guardian. 2015.

[18] Livingstone E, Shepherd R (2016). Bear farms in Lao PDR expand illegally and fail to conserve wild bears. Oryx.

[19] Shairp R, Verissimo D, Fraser I, Challender D, MacMillan D (2016). Understanding urban demand for wild meat in Vietnam: implications for conservation actions. PLoS One.

[20] Scotson L. The distribution and status of Asiatic black bear Ursus thibetanus and Malayan sun bear Helarctos malayanus in Nam Et Phou Louey National Protected Area: Lao PDR. Free the Bears; 2010.

[21] Interpol. 2014. Assessment on illegal bear trade*.* International criminal police organization (ICPO) – INTERPOL.

[22] Wildlife Friends Foundation Thailand (WFFT). 2016. *Laos intends to shut down tiger farms and bear bile farms*. Accessed 20 May 2017. URL: https://www.wfft.org/wildlife-trade/laos-intends-shut-tiger-farms-bear-bile-farms/

[23] Free the Bears (FTB) (2016). *Bears’ print*.

[24] The Provincial Agriculture and Forestry Office of Luang Prabang and Free the Bears (PAFOLP and FTB). 2016. Luang Prabang Wildlife Rescue Centre. Publicity leaflet.

[25] Browne-Nuñez C, Jonker SA (2008). Attitudes toward wildlife and conservation across Africa: a review of survey research. Hum Dimens Wildl.

[26] Govan H, Inglis A, Pretty J, Harrison M, Wightman A. Best practice in community participation for national parks; 1998. Scottish Natural Heritage

[27] Holmes G (2013). Exploring the relationship between local support and the success of protected areas. Conserv Soc.

[28] Walpole MJ, Goodwin HJ (2001). Local attitudes towards conservation and tourism around Komodo National Park, Indonesia. Environ Conserv.

[29] Bennett NJM, Dearden P (2014). Why local people do not support conservation: community perceptions of marine protected area livelihood impacts, governance and management in Thailand. Mar Policy.

[30] Sekhar NU (2003). Local people’s attitudes towards conservation and wildlife tourism around Sariska Tiger Reserve, India. J Environ Manag.

[31] Sirivongs T, Tsuchiya T (2012). Relationship between local residents’ perceptions, attitudes and participation towards national protected areas: a case study of Phou Khao Khouay National Protected Area, central Lao PDR. Forest Policy Econ.

[32] Tessema ME, Lilieholm RJ, Ashenafi ZT, Leader-Williams N (2010). Community attitudes toward wildlife and protected areas in Ethiopia. Soc Nat Resour.

[33] Thapa K (2016). Park – people interaction and public perceptions towards Parsa wildlife reserve, Nepal. Northwestern J Int Law Bus.

[34] Oldekop JA, Holmes G, Harris WE, Evans KL (2015). A global assessment of the social and conservation outcomes of protected areas. Conserv Biol.

[35] Call E. Mending the web of life: Chinese medicine and species conservation. Yarmouth Port: International Fund for Animal Welfare; 2006.

[36] Karanth KK, Randall AK, Song SQ, Norman LC (2008). Examining conservation attitudes, perspectives, and challenges in India. Biol Conserv.

[37] Ewert A, Galloway G (2004). Expressed environmental attitudes and actual behavior: exploring the concept of environmentally desirable responses.

[38] Glasman LR, Albarracín D (2006). Forming attitudes that predict future behavior: a meta-analysis of the attitude-behavior relation. Psychol Bull.

[39] Kioko J, Kiring JW. Youth’s knowledge, attitudes and practices in wildlife and environmental conservation in Maasailand, Kenya, Southern African. J Environ Educ. 2010;27:91–101.

[40] Newing H (2011). Conducting research in conservation.

[41] IBM Corp (2013). IBM SPSS Statistics for Windows, Version 22.0.

[42] Agresti A (2002). Categorical data analysis. 2nd ed.

[43] Chauhan NPS (2003). Human casualties and livestock depredation by black and brown bears in the Indian Himalaya, 1989-98. Ursus.

[44] Charoo SA, Sharma LK, Sathyakumar S (2009). Asiatic Black Bear – human conflicts around Dachigam National Park, Kashmir.

[45] Scotson L, Vannachomchan K, Sharp T (2014). More valuable dead than deterred? Crop-raiding bears in Lao PDR. Wildl Soc Bull.

[46] Eriksson M, Sandström C, Ericsson G (2015). Direct experience and attitude change towards bears and wolves. Nordic Board for Wildlife Res.

[47] Pinheiro L, Rodrigues JFM, Borges-Nojosa DM (2016). Formal education, previous interaction and perception influence the attitudes of people toward the conservation of snakes in a large urban center of northeastern Brazil. J Ethnobiol Ethnomed.

[48] Vaughan C, Gack J, Solorazano H, Ray R (2010). The effect of environmental education on schoolchildren, their parents and community members: a study of intergenerational and intercommunity learning. J Environ Educ.

[49] MacGregor F. Inside a bear bile farm in Laos. Telegraph (London). 2010. Available at: https://www.telegraph.co.uk/news/worldnews/asia/laos/7950161/Inside-a-bear-bile-farm-in-Laos.html. Accessed 19 Aug 2010.

[50] Anthony BP, Abonyi S, Terblanche P, Watt A, Pavlinov IY (2011). Towards bridging worldviews in biodiversity conservation: exploring the Tsonga concept of *ntumbuloko* in South Africa. Research in biodiversity - models and applications.

[51] Howes M, Chambers R (1979). Indigenous technical knowledge: analysis, implications and issues.

[52] Vongvichith E. Case study: Laos-Ministry of Health. Fujitsu. Available at: http://www.fujitsu.com/cn/en/Images/CS_Laos_MOH_en.pdf. 2013; Date accessed: 31 Dec 2017.

[53] Quyen TV (2010). An analysis of attitudes and bear bile use in Vietnam.

[54] Doyle-Capitman CE, Siemer WF, Decker DJ (2017). Revealing the pro-conservation impacts of participation in nature-dependent activities on a local national wildlife refuge.

[55] Suswarndany D, Sibbritt DW, Supardi S, Chang S, Adams J (2015). A critical review of traditional medicine and traditional healer use for malaria and among people in malaria-endemic areas: contemporary research in low to middle-income Asia-Pacific countries. Malar J.

[56] Sydara K, Gneunphonsavath S, Wahlström R, Freudenthal S, Houamboun K, Tomson G (2005). Use of traditional medicine in Lao PDR. Complement Ther Med.

[57] Gavin MC, Solomon JN, Blank SG (2010). Measuring and monitoring illegal use of natural resources. Conserv Biol.

[58] Alves RRN, Rosa IL, Santana GG (2007). The role of animal-derived remedies as complementary medicine in Brazil. BioScience.

[59] Lao Statistics Bureau (LSB), Ministry of Planning and Investment (2015). Results of population and housing census 2015.

